# Crucial role of temporary airborne infection isolation rooms in an intensive care unit: containing the COVID-19 outbreak in South Korea

**DOI:** 10.1186/s13054-020-02944-0

**Published:** 2020-05-18

**Authors:** Shin Yup Lee, Sun Ha Choi, Ji Eun Park, Soyoon Hwang, Ki Tae Kwon

**Affiliations:** 1 Division of Pulmonary and Critical Care Medicine, Kyungpook National University Chilgok Hospital, Daegu, Korea; 2 Department of Internal Medicine, School of Medicine, Kyungpook National University, Daegu, Korea; 3 Division of Infectious Diseases, Kyungpook National University Chilgok Hospital, 807 Hokuk-ro, Buk-gu, Daegu, 41404 Korea; 4 Department of Infection Control, Kyungpook National University Chilgok Hospital, Daegu, Korea

From 20 February through 9 March 2020, South Korea reported the highest number of patients confirmed with coronavirus disease 2019 (COVID-19) outside China, mainly in the Daegu city area: this was a disease cluster related to the practices of the Shincheonjii religious group [1–3]. In Daegu city, the number of patients with COVID-19 increased rapidly and saturated the healthcare system; as a result, some critically ill patients were unable to obtain hospital care and as a result died of this disease. Care for critically ill COVID-19 patients requires intensive care units (ICUs) that are equipped with airborne infection isolation rooms (AIIRs) [4, 5]. To cope with this situation, we assembled temporary AIIRs with mobile negative-air machines in our ICU, as was done previously in Korea during the Middle East Respiratory Syndrome (MERS) outbreak [6]. Here, we share our experiences with the assembly of temporary AIIRs in the ICU of our single 635-bed tertiary care, academic hospital and discuss the critical role played by these units toward controlling this explosive outbreak. We hope that our findings will serve as a reference for areas where the COVID-19 outbreak remains ongoing.

Before the COVID-19 outbreak, Daegu had only three AIIRs with anteroom in an ICU of one national university hospital; these were built with government support after the 2015 MERS outbreak. The tertiary hospitals in Daegu facilitated the isolation and treatment severely ill patients via temporarily remodeling of existing ICU facilities. Prior to the outbreak, our hospital operated two separate ICU facilities; each was equipped with two AIIRs without anterooms. This was not sufficient for isolation of COVID-19 patients nor did this provide appropriate protection for healthcare workers (HCWs). We decided to remodel one of the two ICUs to facilitate isolation of critically ill COVID-19 patients. Schematic view of the renovated ICU was illustrated in Fig. 1. Critically ill COVID-19 patients were transported to the temporary negative pressure isolation ICU in negative pressure carts that utilized an exclusive path and elevator maintained for this purpose by staff members wearing appropriate personal protective equipment. The COVID-19 team included 5 physicians and 40 nurses. All HCWs in our newly remodeled ICU were screened for COVID-19 via the reverse transcriptase-polymerase chain reaction test after the first 2 weeks on duty; no tests were reported as positive.

**Fig. 1:**
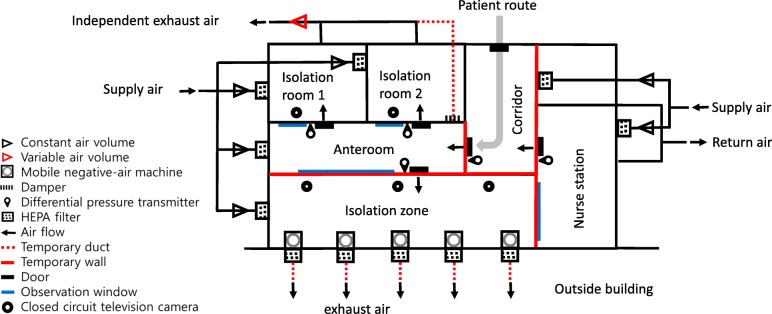
Schematic view of the temporary negative pressure isolation intensive care unit. We divided the space to include a common anteroom, a common negative pressure isolation zone to accommodate three beds, two preexisting airborne infection isolation rooms (AIIRs), and a nursing station. Atmospheric air was supplied to the ICU including AIIRs via a mechanism that maintained constant air volume through common inlet duct systems. The air returned from ICU through common outlet duct systems; the AIIRs utilized independent exhaust systems that were controlled by a variable air volume system to maintain a set negative pressure. To generate negative pressure in the anteroom, we added temporary duct systems that were connected to preexisting independent exhaust systems. An air volume control damper was used to maintain a negative pressure gradient between preexisting AIIRs and the anteroom (− 5.0 Pa) at a level below the standard negative pressure (− 2.5 Pa) recommended for these facilities. The common negative pressure isolation zone was equipped with five mobile negative-air machines that generated negative pressure (− 5.0 Pa) compared to the anteroom. Airflow in isolation rooms reached 15–20 air exchanges per hour. The negative pressure in the ICU was tightly monitored and maintained. Patients were monitored via an observation window, closed-circuit television, and central monitoring systems. The entire renovation was completed within 5 days

Approximately 60% of the COVID-19 patients at our hospital had severe pneumonia (respiratory rate > 30 breaths/min, severe respiratory distress, or SpO_2_ ≤ 93% on room air). Before the AIIRs in the ICU opened in our hospital, we had no choice but to treat these patients in preexisting AIIRs located with the general hospital ward. Upon completion of this renovation, we were able to hospitalize all critically ill COVID-19 patients and to provide timely and appropriate management and support. Our newly renovated temporary AIIRs have been in operation for 4 weeks. During this time, we treated seven patients: six patients have required mechanical ventilation, two patients were treated with extracorporeal membrane oxygenation, and continuous renal replacement therapy has been provided to one patient. We believe that the urgently renovated ICU played an important role in preventing the surge of mortality despite the rapid increasing number of patients with severe pneumonia (Fig. 2).

**Fig. 2:**
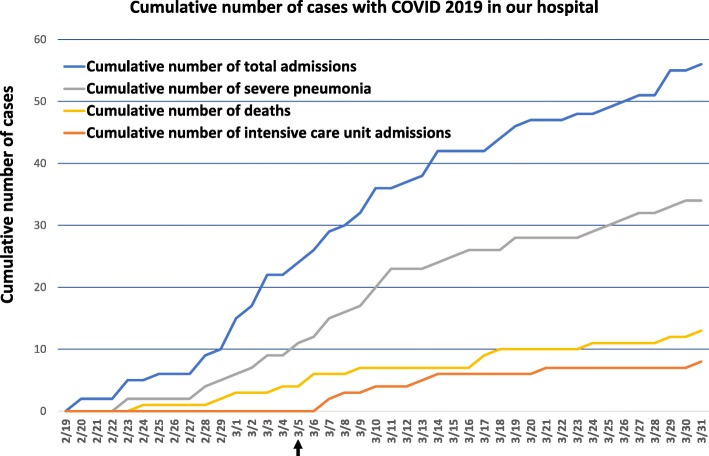
The cumulative number of cases with coronavirus disease 2019 (COVID-19) in our hospital. On March 5, 2020 (arrow), the remodeling of intensive care unit (ICU) for critically ill patients with COVID-19 was completed. This critical renovation permitted us to manage critically ill COVID-19 patients at our hospital

In conclusion, our experience suggests that renovation of our ICUs to include temporary AIIRs was a critical and highly effective measure that permitted us to react appropriately to the explosive outbreak of COVID-19 and to provide optimal care for severely ill patients. We hope that our experience will help to prepare physicians and hospitals worldwide for the unprecedented crisis of the COVID-19 pandemic.

## Data Availability

The data are available from the corresponding author.

## Abbreviations

COVID-19: Coronavirus disease 2019
ICU: Intensive care unit
AIIRs: Airborne infection isolation rooms
MERS: Middle East Respiratory Syndrome
HCWs: Healthcare workers
